# Seasonality Affects the Phenolic Composition and Erythroprotective Activity of Ora‐Pro‐Nobis (*Pereskia aculeata*) Leaves

**DOI:** 10.1002/cbdv.202502379

**Published:** 2025-12-19

**Authors:** Thiago Mendanha Cruz, Cristiane de Moura, Daniel Granato, Mariza Boscacci Marques

**Affiliations:** ^1^ Graduation Program in Chemistry State University of Ponta Grossa, Av. Carlos Cavalcanti Ponta Grossa Brazil; ^2^ Department of Food Science and Microbiology Auckland University of Technology Auckland New Zealand; ^3^ AUT Centre for Future Foods Auckland University of Technology Auckland New Zealand

**Keywords:** antioxidant capacity, Cactaceae, harvest time, oxidative stress, protein oxidation

## Abstract

Ora‐pro‐nobis (OPN) is a food plant with polyphenol‐rich leaves. However, the influence of seasonality on their quantitative phenolic composition and how it impacts their bioactivities is unknown. OPN leaves were harvested throughout 12 months, and their individual phenolic content, antioxidant activity, and erythroprotection were assessed. The highest total phenolic content was recorded in September (736 mg GAE/100 g), and the lowest in October (104 mg GAE/100 g). September also exhibited the highest rutin and protocatechuic, *p*‐coumaric, and caffeic acids concentrations. Rutin was the major compound throughout the year. Protocatechuic acid contents were correlated with the air relative humidity, solar radiation incidence, and air temperature. June sample presented the best 2,2‐diphenyl‐1‐picrylhydrazyl scavenging activity (227 mg AAE/100 g), while August was the most efficient sample to Fe^2+^‐chelating (450 mg EDTAE/100 g), and September exhibited the highest ferric reducing antioxidant power (285 mg AAE/100 g). The December sample showed the greatest protection of erythrocytes against osmotic stress (48% haemolysis). Under oxidative conditions, November extract reduced the most lipoperoxidation and haemoglobin oxidation, and July was the most effective against haemolysis. Thus, seasonality appears to be a crucial factor in the biosynthesis of bioactive compounds in OPN leaves, which alters their biological properties but not their safety for consumption.

## Graphical abstract

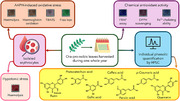

## 1 Introduction

In front of the challenge of providing food for the world population in the climate change era, when mass food production from monocultures and animal sources has been questioned due to their environmental impact, unconventional food plants (UFPs) have arisen on the horizon as an alternative food source. UFPs are plants with at least one edible part that are not widely consumed, despite being known, consumed, and cultivated especially by traditional communities and family farmers [1]. Among these UFPs, a Cactaceae food plant popularly known as ora‐pro‐nobis (OPN) has stood out.

OPN (*Pereskia aculeata* Mill.) leaves are protein‐, carbohydrate‐, and polyphenolic‐rich [2]. OPN has gained attention from the food industry, and some food additives have been developed from these leaves, aiming to improve the nutritional value and functional properties of other foods [2, 3]. In addition to their nutritional importance, the intake of OPN leaves may confer health benefits. Anti‐inflammatory, neuroprotective, antioxidant, antimicrobial, and antihaemolytic activities are properties already scientifically attributed to OPN leaf extracts and these bioactivities are often related to polyphenols [4, 5, 6].

Phenolic compounds already identified in OPN leaves include flavonoids, stilbenes, and hydroxycinnamic and hydroxybenzoic acids [2, 7, 8]. Quantifying these compounds is remarkable since several environmental factors may influence the biosynthesis of polyphenols by the plant, and, for Cactaceae plants, seasonality is a key aspect that rules this production [9]. However, 56 different compounds, including polyphenolic compounds, were reported in OPN harvested during the winter and autumn, but 54 of these substances were present in both harvest times [7]. Nonetheless, limited quantitative data regarding phenolic composition throughout the year are found in the literature, which is required to elucidate the impact of seasonality on the expected biological properties and toxicity, especially the cellular protection against abiotic stresses.

In this sense, human red blood cells (RBCs, or erythrocytes) are an appropriate biological model to investigate the cellular protective capacity of bioactive compounds since the assays using RBCs are quick, inexpensive, and easy to run. When under stress, erythrocytes are damaged, causing haemolysis and biomolecule oxidation and leading to functional loss [10]. OPN leaf extracts are reported as effective RBC protectors against oxidative and osmotic stresses [2, 5, 6], but the influence of the changes in chemical composition provoked by seasonality on this protective effect has still been ignored.

Considering that there is a gap in the quantitative variation of polyphenol content depending on the harvest time and its consequent impact on the bioactivities of OPN, the objective of this work was to evaluate the influence of seasonality on the phenolic composition, antioxidant capacity, and antihaemolytic activity of OPN. We hypothesise that (1) the phenolic composition is changeable throughout the year, (2) the harvest time influences the in vitro antioxidant and antihaemolytic properties, and (3) the most significant protective effect will be verified in the samples with the highest polyphenol levels.

## 2 Results and Discussion

### 2.1 Phenolic Composition

The samples’ total phenolic content (TPC) ranged from 104 to 736 mg GAE/100 g of leaves (Table 1), lower than the 992–4375 mg GAE/100 g reported before for OPN leaves [11]. The highest TPCs were noted during the quiescence and new leaves stages. These results do not corroborate the findings previously reported for OPN leaves, that the greatest TPC occurs during the flowering period, and the lowest levels in February, right after the formation of new leaves [12].

**TABLE 1:** Total and individual phenolic composition obtained by high‐performance liquid chromatography/diode array detection/ultraviolet (HPLC/DAD/UV) of ora‐pro‐nobis leaf extracts throughout the year.

Sample	TPC (mg GAE/100 g)	Gallic acid (µg/g)	Protocatechuic acid (µg/g)	Caffeic acid (µg/g)	*p*‐Coumaric acid (µg/g)	Ferulic acid (µg/g)	Rutin (µg/g)	Quercetin (µg/g)
**January**	443 ± 11^d^	<LOQ	66 ± 3^g^	76 ± 4^e^	<LOQ	30 ± <1^b^	632 ± 63^b^	ND
**February**	117 ± 3^h^	21 ± 1^b^	23 ± <1^h^	20 ± <1^g^	<LOQ	ND	128 ± 9^fg^	ND
**March**	371 ± 15^e^	<LOQ	77 ± 1^ef^	126 ± 1^a^	<LOQ	53 ± 6^a^	219 ± 17^de^	ND
**April**	443 ± 12^d^	<LOQ	88 ± 1^d^	89 ± 3^d^	<LOQ	27 ± <1^b^	406 ± 36^c^	ND
**May**	327 ± 4^f^	<LOQ	84 ± <1^de^	87 ± 1^d^	ND	34 ± 3^b^	416 ± 14^c^	16 ± <1^a^
**June**	505 ± 21^c^	<LOQ	122 ± 6^c^	ND	10 ± 1^d^	29 ± 1^b^	148 ± 3^g^	ND
**July**	386 ± 6^e^	<LOQ	132 ± 1^b^	73 ± <1^e^	30 ± 1^c^	ND	190 ± 7^ef^	ND
**August**	560 ± 9^b^	<LOQ	122 ± 1^c^	109 ± 2^b^	178 ± 19^b^	28 ± <1^b^	268 ± 25^d^	15 ± <1^a^
**September**	736 ± 2^a^	<LOQ	147 ± <1^a^	122 ± 1^a^	233 ± 25^a^	ND	816 ± 34^a^	ND
**October**	104 ± 10^h^	ND	22 ± <1^h^	ND	ND	ND	105 ± 3^g^	ND
**November**	267 ± 10^g^	56 ± <1^a^	76 ± 1^f^	40 ± <1^f^	ND	17 ± 1^c^	129 ± 1^fg^	ND
**December**	466 ± 21^d^	<LOQ	63 ± 1^g^	100 ± 1^c^	<LOQ	24 ± 3^c^	235 ± 17^de^	12 ± <1^b^
*p*‐value homoscedasticity	0.886	0.489	0.445	0.635	0.951	0.375	0.225	0.278
*p*‐value ANOVA	≤0.05	≤0.05 (paired *t*‐test)	≤0.05	≤0.05	≤0.05	≤0.05	≤0.05	≤0.05

*Note*: ND = not detected; LOQ = limit of quantification; NA = not applicable; GAE = gallic acid equivalents. Different letters in the same column represent statistically different results (*p* ≤ 0.05).

Gallic acid, rutin, protocatechuic acid, ferulic acid, caffeic acid, *p*‐coumaric acid, and quercetin were previously identified in OPN leaves [2, 7, 8], and, herein, were utilised as chemical markers to understand the impact of seasonality on the chemical composition of OPN leaves (Table 1, Figure S3). Quercetin was detected only in the May, August, and December samples, with contents ranging from 12 to 16 µg/g, lower than the previously reported (31.7–48.9 µg/g) [13]. The presence of quercetin is also not constant throughout the year for other plants. The *Senna singueana* (Del.) Lock leaves, for example, only present this flavonoid in their composition during the Summer [14]. On the other hand, rutin was found in all our samples, being the major compound with contents varying between 105 and 816 µg/g, which agrees with what was found in the literature [15]. As found in our samples, the peak of rutin production reported in *Moringa oleifera* Lam. leaves was in winter [16]. Gallic acid was quantified only in the February and November samples (21 and 56 µg/g, respectively), the months that present the greatest air temperature average (Table S2), and these contents were greater than the formerly reported (4.7–6.2 µg/g) [7].

**FIGURE 1:**
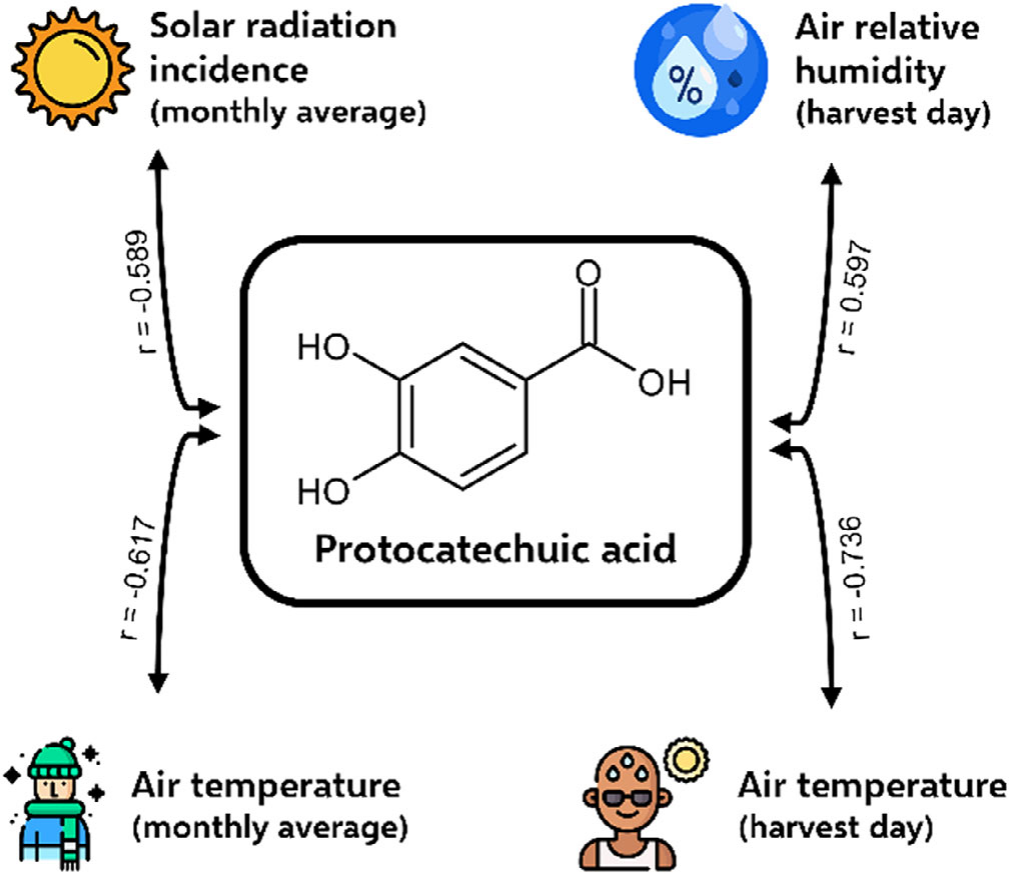
Significative (p ≤ 0.05) Pearson correlations between protocatechuic acid contents in ora‐pro‐nobis leaves and meteorological parameters.

Protocatechuic acid was present in OPN leaves throughout the year, with 22–147 µg/g as content. Its biosynthesis was increased during the quiescence stage. This phenolic acid was formerly quantified in *Pereskia grandifolia* Haw. leaves, and the contents reported were 14–34 µg/g extract [17]. Protocatechuic acid was the only compound whose contents were statistically correlated with environmental parameters: solar radiation incidence and air temperature, and relative humidity (Figure 1). These correlations are a preliminary indication of how to maximise its content in OPN leaves: high air relative humidity and low solar radiation incidence, and air temperature. Some hydroxybenzoic acids, i.e., protocatechuic acid, are linked to water absorption deficiency in plants [18], which may justify the direct correlation with the air's relative humidity, since when the amount of available water in the air is wispy, the plant can reduce the biosynthesis of protocatechuic acid to avoid dehydration.

*p*‐Coumaric acid levels were increased during the quiescence period, with contents ranging between 10 and 233 µg/g, greater than the literature reports (19–28 µg/g) [13]. Caffeic acid was not detected in the October and June samples, but in other samples, its contents (20–126 µg/g) were lower than the 176 µg/g previously reported for OPN leaves [2]. In our OPN samples, the greatest content was recorded during the Summer, similar to the reported for *M. oleifera* leaves [19]. Ferulic acid contents varied between 17–53 µg/g, similar to the formerly found for OPN leaves (25–54 µg/g) [13]. Herein, the greatest ferulic acid content was recorded in March (summer end, flowering stage), which is interesting since this month was the one with the highest solar irradiation average and one of the driest of the analysed period; curiously, this ferulic acid is utilised by several plants to signal transpiration reduction, preventing water loss [18].

The total and individual phenolic contents are variable throughout the year in several plants and plant products, including leaves [20], fruits [21], bracts [22], stem barks [14], etc. Several aspects may provoke these seasonal changes, including climate and phenological factors. It occurs because the secondary metabolites are biosynthesised to respond to environmental stimuli, and these stimuli may be of biotic (microbial infection) or abiotic (i.e., temperature, radiation, water availability, and pluviosity) origin [23].

### 2.2 Antioxidant Activity

**FIGURE 2:**
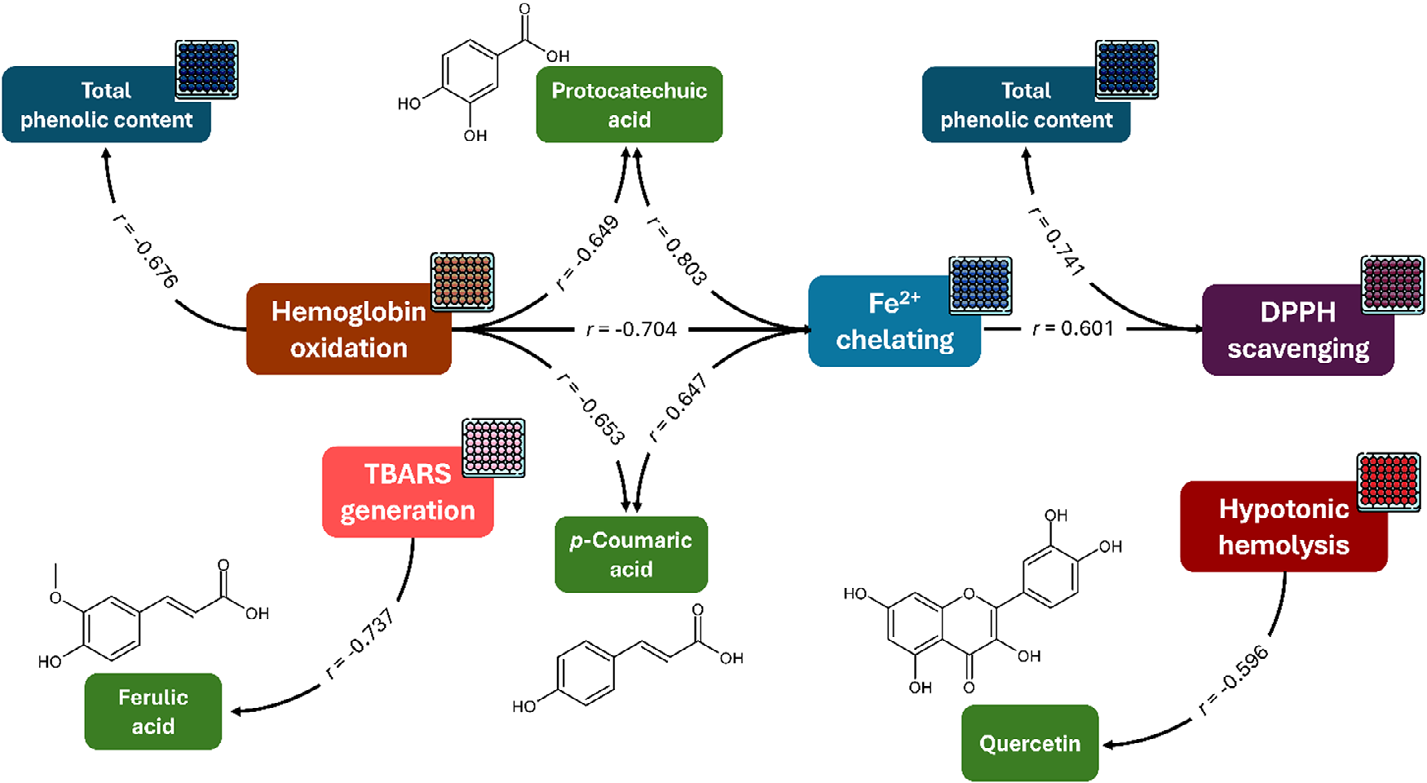
Significative (*p* ≤ 0.05) Pearson correlations between phenolic compounds contents and antioxidant and antihaemolytic activities of ora‐pro‐nobis leaves.

The OPN leaves were effective in the 2,2‐diphenyl‐1‐picrylhydrazyl (DPPH) radical scavenging (Table 2, Figure S4), with an efficiency that varied between 42 (February) and 227 mg AAE/100 g (June). The antioxidant capacity to inhibit the DPPH radical of OPN leaves was statistically correlated (*p* ≤ 0.05) with their total phenolic compound content (Figure 2), indicating that the polyphenol‐rich the leaves, the greater their antioxidant activity. Not coincidentally, it is widely known that phenolic compounds are strong antioxidant compounds [4]. Previously, the half‐maximal inhibitory concentration (IC_50_) reported ranged from 306 to 7829 µg/mL for OPN leaf extracts [24]. Unlike our results for OPN, the moringa leaf samples showed consistent DPPH‐inhibiting antioxidant capacity throughout the year, except for the sample collected during autumn, which showed less activity than the others [19].

**TABLE 2:** Seasonal variation of the chemical antioxidant potential and erythrocytes protection against osmotic and oxidative stresses by ora‐pro‐nobis leaf extract.

Sample	Chemical antioxidant activity			Hypotonic stress		AAPH‐induced stress			
	DPPH^*^	FRAP^*^	Fe^2+^ chelating ability^**^	H_50_ (%)	Haemolysis [NaCl] = 0.4% (%)	Haemolysis (%)	TBARS (%)	Haemoglobin oxidation (%)	Free iron (%)
**January**	140 ± 12^bc^	160 ± 4^c^	184 ± 5^f^	0.412 ± 0.001^b^	58 ± 5^cd^	14 ± 2^fg^	62 ± 5^d^	30 ± 3^b^	50 ± 1^cde^
**February**	24 ± 1^g^	46 ± 3^g^	83 ± 4^g^	0.416 ± 0.003^ab^	66 ± 5^bc^	35 ± 5^d^	73 ± 2^c^	22 ± 1^c^	54 ± 1^bc^
**March**	144 ± 14^bc^	181 ± 2^b^	165 ± 6^f^	0.409 ± 0.009^b^	64 ± 7^cd^	55 ± 5^c^	72 ± 3^c^	24 ± 1^bc^	55 ± 2^bc^
**April**	152 ± 8^b^	143 ± 9^cd^	292 ± 18^d^	0.416 ± 0.005^ab^	69 ± 4^bc^	25 ± 2^e^	61 ± 5^d^	25 ± 4^bc^	48 ± 2^de^
**May**	71 ± 3^e^	99 ± 1^e^	319 ± 2^cd^	0.406 ± 0.001^b^	55 ± 3^cd^	27 ± 3^e^	95 ± 1^a^	27 ± 2^b^	56 ± 1^b^
**June**	227 ± 10^a^	66 ± 5^f^	359 ± 23^c^	0.410 ± 0.001^b^	60 ± 1^cd^	63 ± 6^bc^	43 ± 1^f^	24 ± 1^bc^	45 ± 2^e^
**July**	99 ± 2^d^	130 ± 1^d^	249 ± 1^e^	0.402 ± 0.002^b^	54 ± 8^cd^	10 ± 2^g^	51 ± 3^e^	29 ± 4^b^	48 ± 5^de^
**August**	119 ± 15^cd^	194 ± 13^b^	450 ± 11^a^	0.407 ± 0.006^b^	57 ± 4^cd^	43 ± 1^d^	65 ± 1^d^	24 ± 2^bc^	50 ± 2^cde^
**September**	148 ± 16^bc^	285 ± 9^a^	404 ± 24^b^	0.412 ± 0.005^b^	63 ± 5^cd^	55 ± 6^c^	50 ± 2^e^	22 ± 1^dc^	54 ± 2^bcd^
**October**	42 ± 1^f^	40 ± 2^g^	80 ± 5^g^	0.415 ± 0.003^b^	65 ± 8^c^	22 ± 4^efg^	75 ± 3^c^	27 ± 1^b^	47 ± 2^e^
**November**	98 ± 5^d^	102 ± 2^e^	186 ± 13^f^	0.407 ± 0.001^b^	59 ± 10^cd^	25 ± 2^ef^	40 ± 3^f^	20 ± 1^c^	46 ± 4^e^
**December**	174 ± 17^b^	114 ± 10^e^	333 ± 20^c^	0.401 ± 0.004^b^	48 ± 1^d^	75 ± 7^b^	83 ± 1^b^	20 ± <1^e^	54 ± 2^bcd^
**Quercetin** ^***^	—	—	—	—	53 ± 3^d^	32 ± 5^de^	62 ± 4^d^	39 ± 3^a^	47 ± <1^e^
**Positive control**	—	—	—	—	—	89 ± 3^a^	100 ± 2^a^	43 ± 5^a^	100 ± 1^a^
**Negative control**	—	—	—	0.430 ± 0.003^a^	89 ± 2^a^	2 ± <1	20 ± 1^g^	26 ± 4^bc^	18 ± 1^f^
*p*‐value Homoscedasticity	0.693	0.460	0.756	0.105	0.115	0.896	0.857	0.876	0.646
*p*‐value ANOVA	≤0.05	≤0.05	≤0.05	≤0.05	≤0.05	≤0.05	≤0.05	≤0.05	≤0.05

^*^Results expressed as mg AAE/100 g.

^**^Results expressed as mg EDTAE/100 g.

^***^Quercetin concentration = 25 µg/mL.

*Note*: AAE = ascorbic acid equivalents; EDTAE = EDTA equivalents; TBARS = thiobarbituric acid reactive substances. Different letters in the same column represent statistically different results (*p* ≤ 0.05).

The extracts were also efficient in chelating between 80 and 450 mg EDTAE/100 g of dry OPN leaves, with the October sample being the least efficient and August the greatest (Table 2). These values were lower than the 545 mg EDTAE/100 g found in the literature [2]. The samples’ chelating capacity was statistically correlated (*p* ≤ 0.05) with the protocatechuic acid and *p*‐coumaric acid contents (Figure 2), agreeing with previously evidenced for OPN samples [2]. The samples exhibited ferric reducing antioxidant power (FRAP) between 40 and 285 mg AAE/100 g (Table 2). The greatest potential was verified in the sample harvested in September, during the Winter, and the lowest was observed in October, harvested at the beginning of Spring, agreeing with the previously found for *Calligonum polygonoides* L. foliage [25]. These contents were lower than 5890–23423 mg AAE/100 g, values previously verified in purple tea leaves (*Camellia sinensis* var. *assamica* L. Kuntze) [26].

### 2.3 Seasonality Effect on the Erythrocytes Protection Against Osmotic Stress

**FIGURE 3:**
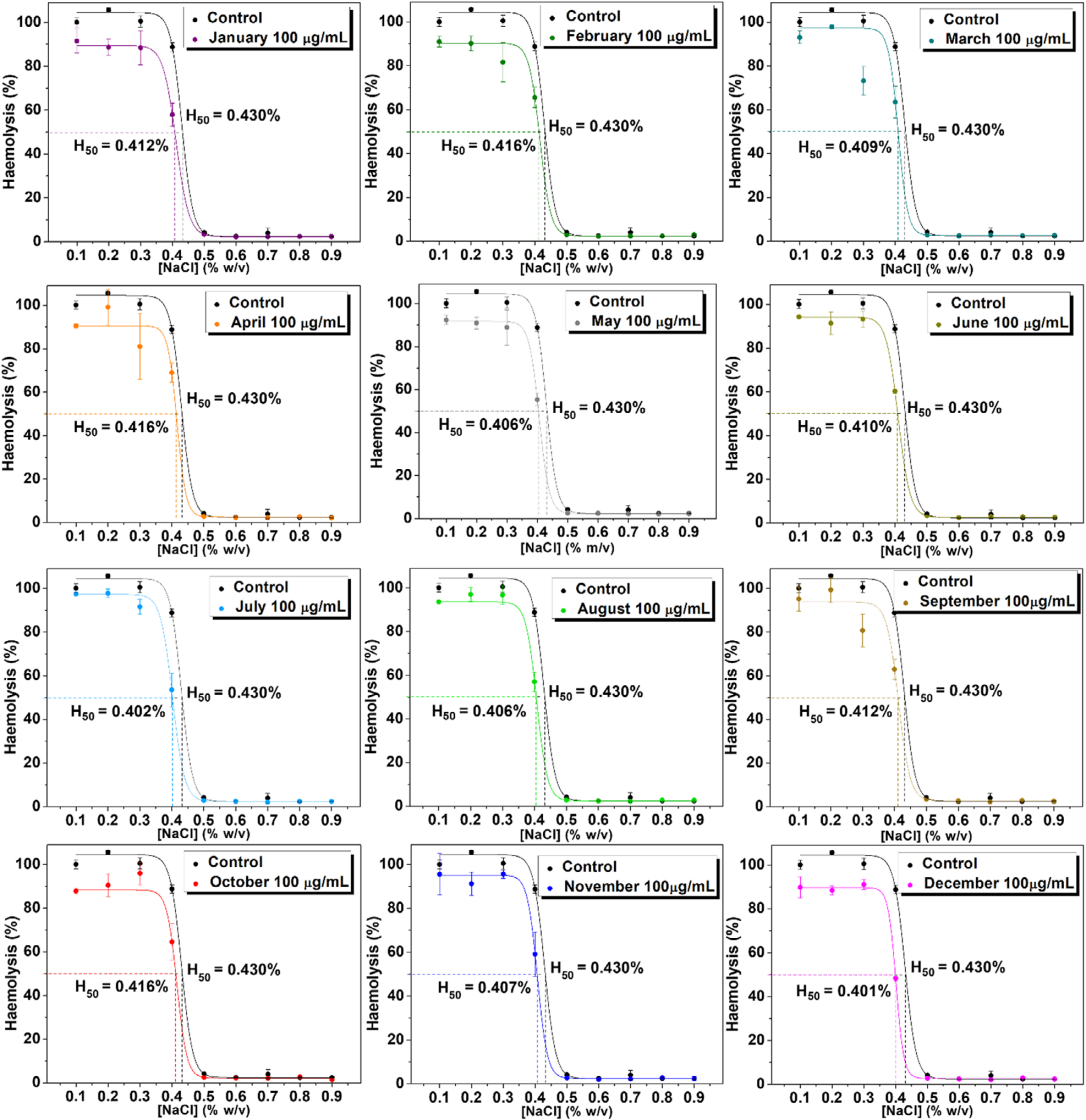
Hypotonic haemolysis curves in the presence of ora‐pro‐nobis (OPN) leaf extracts obtained from samples harvested throughout the year.

**FIGURE 4:**
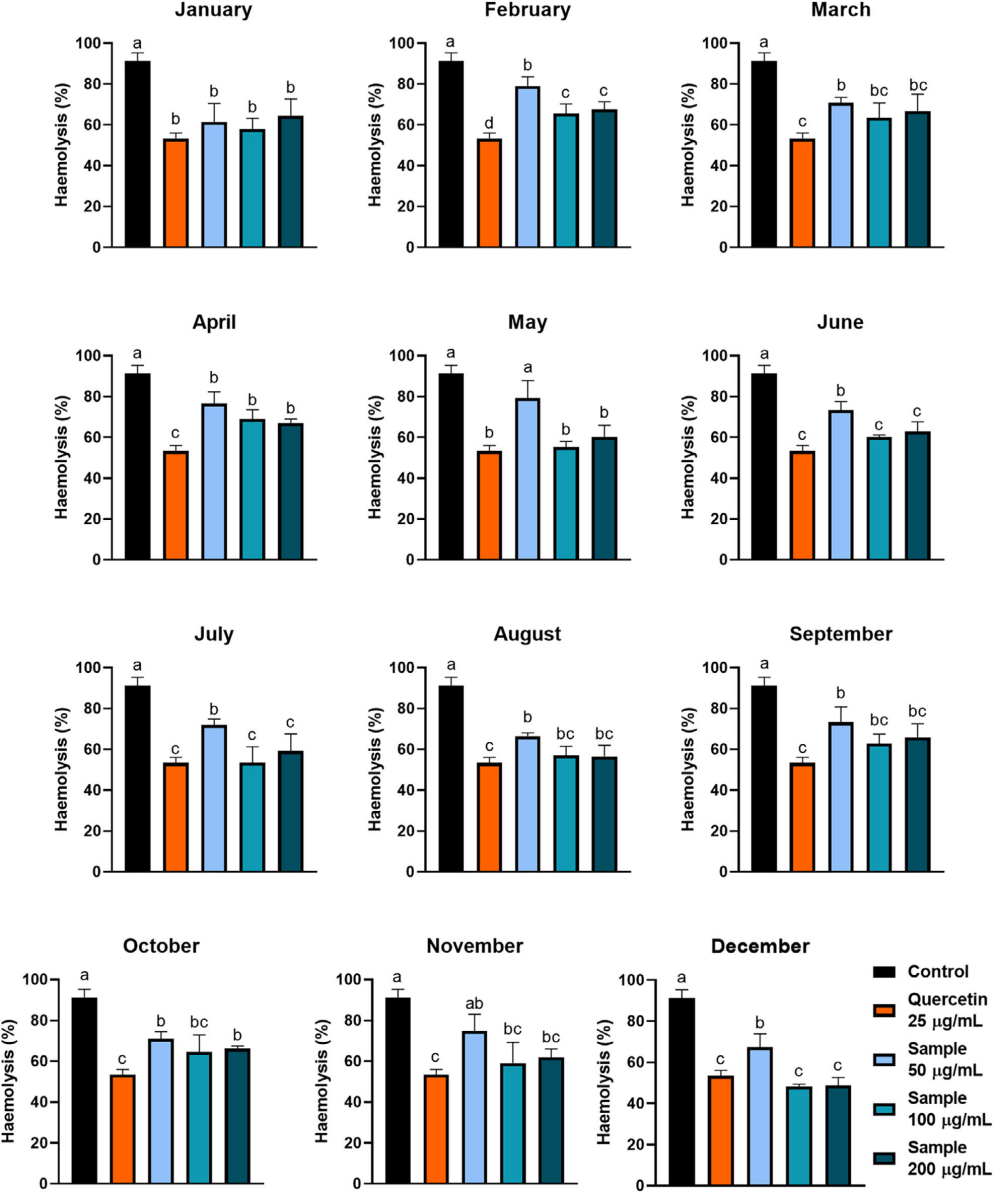
Dose‐dependent effect of ora‐pro‐nobis samples harvested throughout the year on the protection of erythrocytes against osmotic stress ([NaCl] = 0.4%, w/v). Different letters represent statistically different results (*p* ≤ 0.05).

The samples were incubated with RBCs, and these cells were exposed to different osmolarities, from isotonic conditions ([NaCl] = 0.9% w/v) to extreme hypotonic conditions ([NaCl] = 0.1% w/v) (Figure 3 and Table 2). When concentrated at 100 µg/mL, all the extracts (except February and April samples) significantly reduced the H_50_ value, the NaCl concentration at which 50% haemolysis occurs, and these values were not different for each sample. At the NaCl concentration is 0.4%, all extracts significantly reduce the haemolysis rate, with an efficiency similar to that evidenced with quercetin 25 µg/mL (excepting February, April, and October samples). These haemolysis rates were inversely correlated with the samples’ quercetin contents (Figure 2), which is expected since quercetin is known for its antihaemolytic property [27]. Moreover, except for May and November extracts, all the samples inhibit haemolysis at the lowest concentration tested (50 µg/mL), and this inhibition is dose‐dependent for extracts from samples harvested in February, May, June, July, and December (Figure 4). Plant bioactive compounds reduce hypotonic haemolysis by stabilising the erythrocyte membrane and reducing its fluidity [6].

### 2.4 Seasonality Effect on the Erythrocytes Protection Against Oxidative Stress

#### 2.4.1 Oxidative Haemolysis

At 100 µg/mL, all samples diminished the haemolysis rates from 89% (positive control) to 10%–75% (Table 2). Samples harvested in January and July presented the greatest efficacies, being more efficient than quercetin. Despite being the least efficient sample, December was still efficient. February, April, May, August, October, and November extracts showed no different protection from that verified with quercetin. Only the July sample did not exhibit dose‐dependent behaviour, with haemolysis rates varying between 8%–14% (Figure 5A). June, September, and December samples did not significantly reduce the haemolysis rate when concentrated at 50 µg/mL. Moreover, March, September, and December samples were unique whose none of the tested concentrations unveiled a similar or better protection than quercetin. So far, the IC_50_ of an OPN leaf extract revealed in the literature was 57 µg/mL (30 min) and 131 µg/mL (60 min) [5]. Also, purple tea reduced the haemolysis rate from 75% to 20% [28]. Antihaemolytic compounds protect RBCs from haemolysis by preserving their lipids from peroxidation and filling spaces created by the oxidant in the cellular membrane [10].

FIGURE 5(A) Effect of seasonality on the protection of erythrocytes against 2,2'‐Azobis(2‐amidinopropane) dihydrochloride (AAPH)‐induced oxidative haemolysis by ora‐pro‐nobis leaf extracts. (B) Lipoperoxidation inhibition capacity of ora‐pro‐nobis leaves harvested throughout the year on red blood cells. Different letters represent statistically different results (*p* ≤ 0.05).

**Figure:**
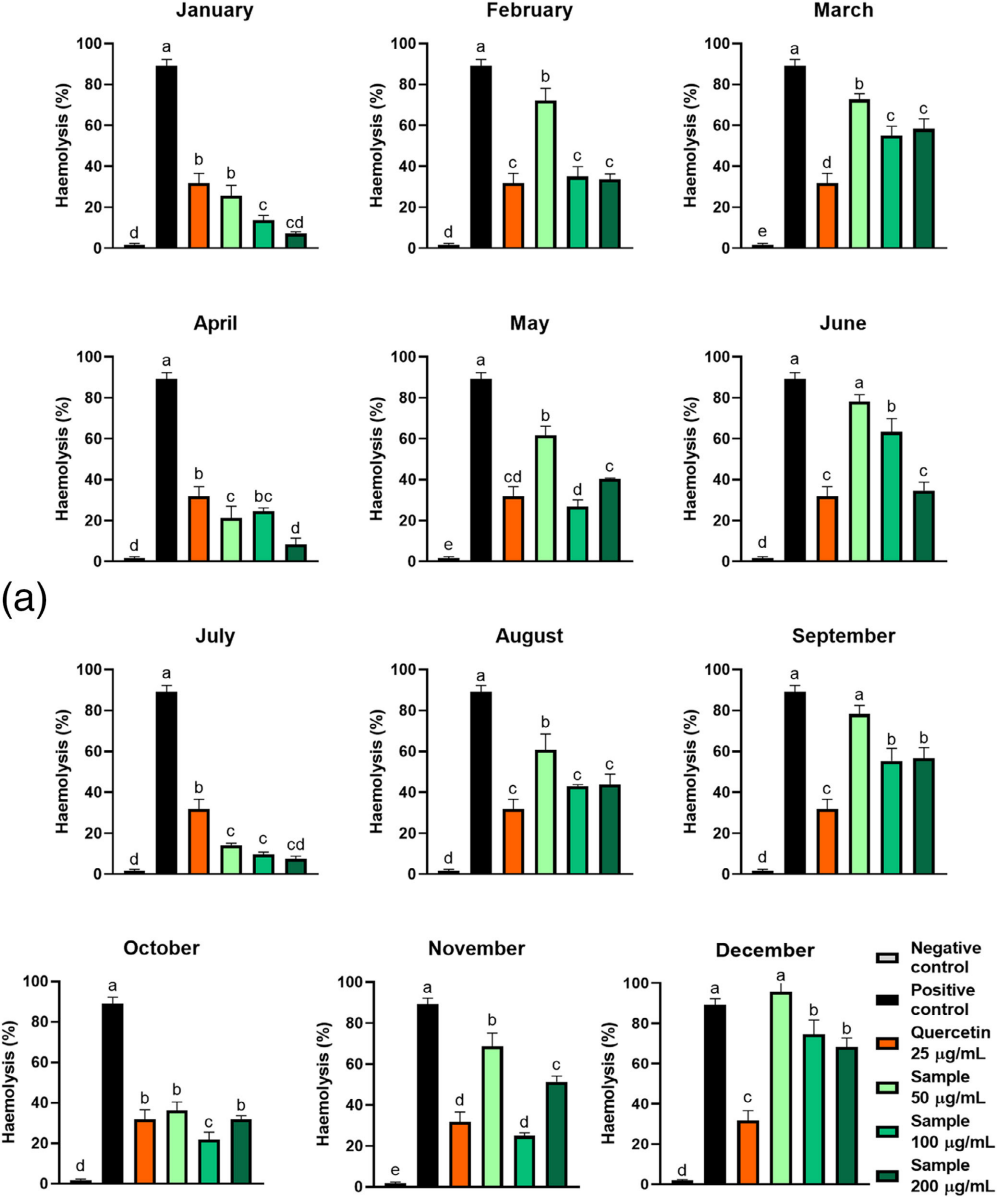

**Figure:**
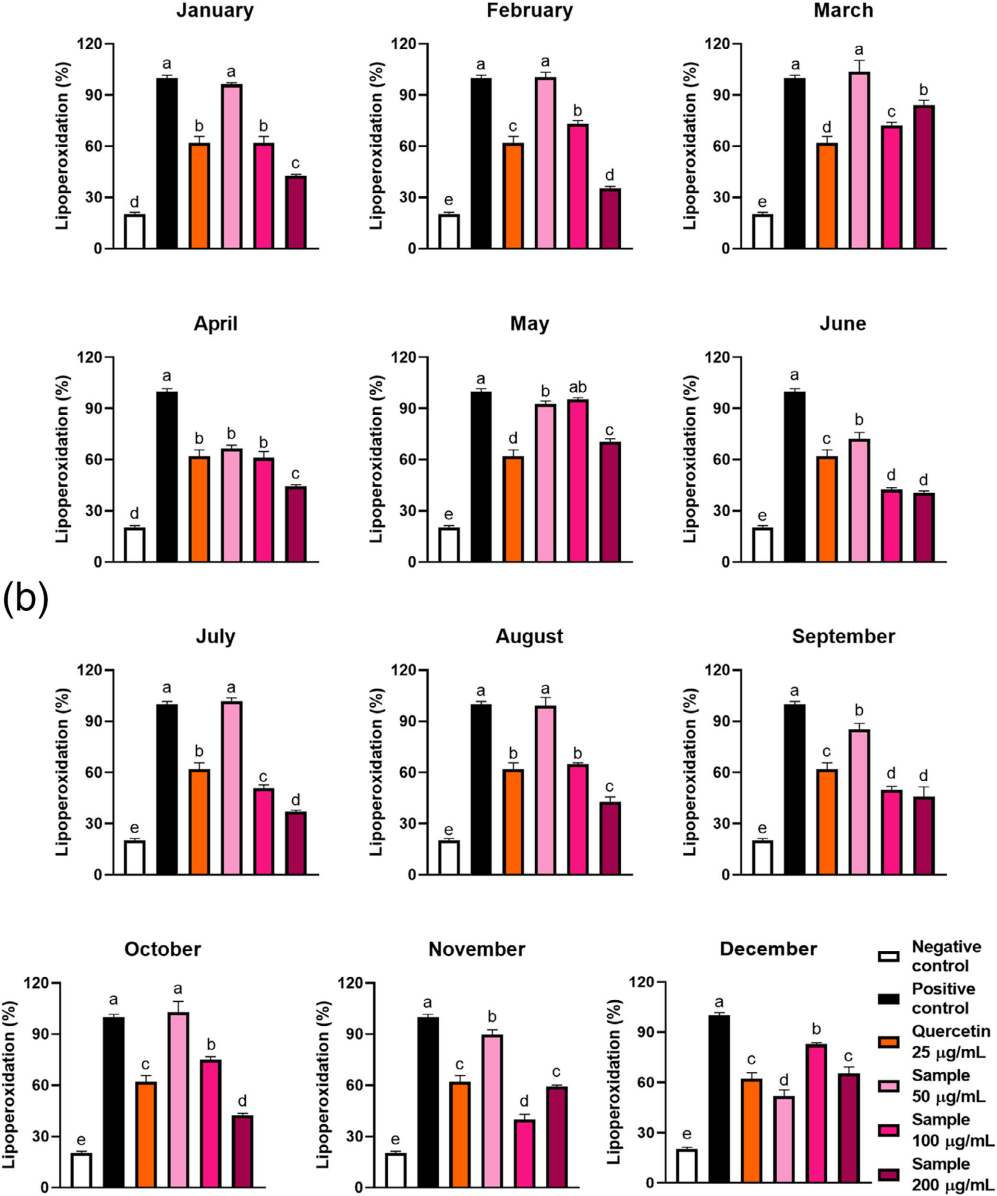

#### 2.4.2 Lipoperoxidation (Thiobarbituric Acid Reactive Substances Assay)

One of the consequences of oxidative stress in RBCs is the peroxidation of their lipids [29]. The reactive species generated during the AAPH‐induced oxidative stress transform polyunsaturated fatty acids into lipid hydroperoxides, which are unstable and quickly decompose to aldehydes and malondialdehydes (MDAs). These by‐products are also called thiobarbituric acid reactive substances (TBARS), since they react with thiobarbituric acid to form a pink complex compound. Bioactive compounds may act to scavenge the reactive species generated by 2,2'‐Azobis(2‐amidinopropane) dihydrochloride (AAPH), protecting lipids from peroxidation [10].

OPN leaf extracts were previously reported as lipid protectors, presenting IC_50_ = 39 µg/mL [5]. At 100 µg/mL (Table 2), the June and November extracts presented the greatest efficiency, while the May sample was not effective in protecting RBC lipids against oxidation. June, July, September, and November samples showed better protection than quercetin 25 µg/mL. Each extract exhibited a dose‐dependent effect on lipoperoxidation inhibition (Figure 5B). At 200 µg/mL, all the samples significantly reduced the TBARS generation, indicating that the extracts protected RBCs against lipoperoxidation. Interestingly, the April, May, June, September, November, and December extracts were also efficacious at the lowest tested concentration (50 µg/mL). Moreover, TBARS generation was inversely correlated to the samples’ ferulic acid content (Figure 2), a well‐known compound for protecting biological matrices against lipoperoxidation [30].

### 2.5 Haemoglobin Oxidation

Oxyhaemoglobin is the functional form of haemoglobin, in which the metallic centre is Fe^2+^. Under oxidative stress, this ion is oxidised to Fe^3+^, changing the protein form to methaemoglobin and causing the RBCs to lose their function [31]. Our samples were effective in protecting haemoglobin against oxidation, diminishing the oxidation rates from 43% (positive control) to 20%–30% (extracts concentrated at 100 µg/mL), and these values were not different from the negative control (Table 2). The samples exhibited efficacy at 50 and 100 µg/mL (Figure 6A); however, January, April, July, and October samples lost their efficiency at 200 µg/mL. Curiously, only the January, April, July, August, and October samples exhibited a dose‐dependent effect. Haemoglobin oxidation rates were inversely correlated with the samples’ TPC, which was expected since phenolic compounds are recognised antioxidants and may protect proteins against oxidation [32]. In the literature, it is possible to find that other leaf matrices effectively reduced haemoglobin oxidation, i.e., *M. oleifera* leaves, which reduced 10‐fold the haemoglobin oxidation in RBC [33].

FIGURE 6(A) Effect of seasonality on the haemoglobin oxidation protection by ora‐pro‐nobis leaf extracts. (B) Free iron rate detected in oxidised erythrocytes incubated with extracts of ora‐pro‐nobis leaves harvested throughout the year. Different letters represent statistically different results (*p* ≤ 0.05).

**Figure:**
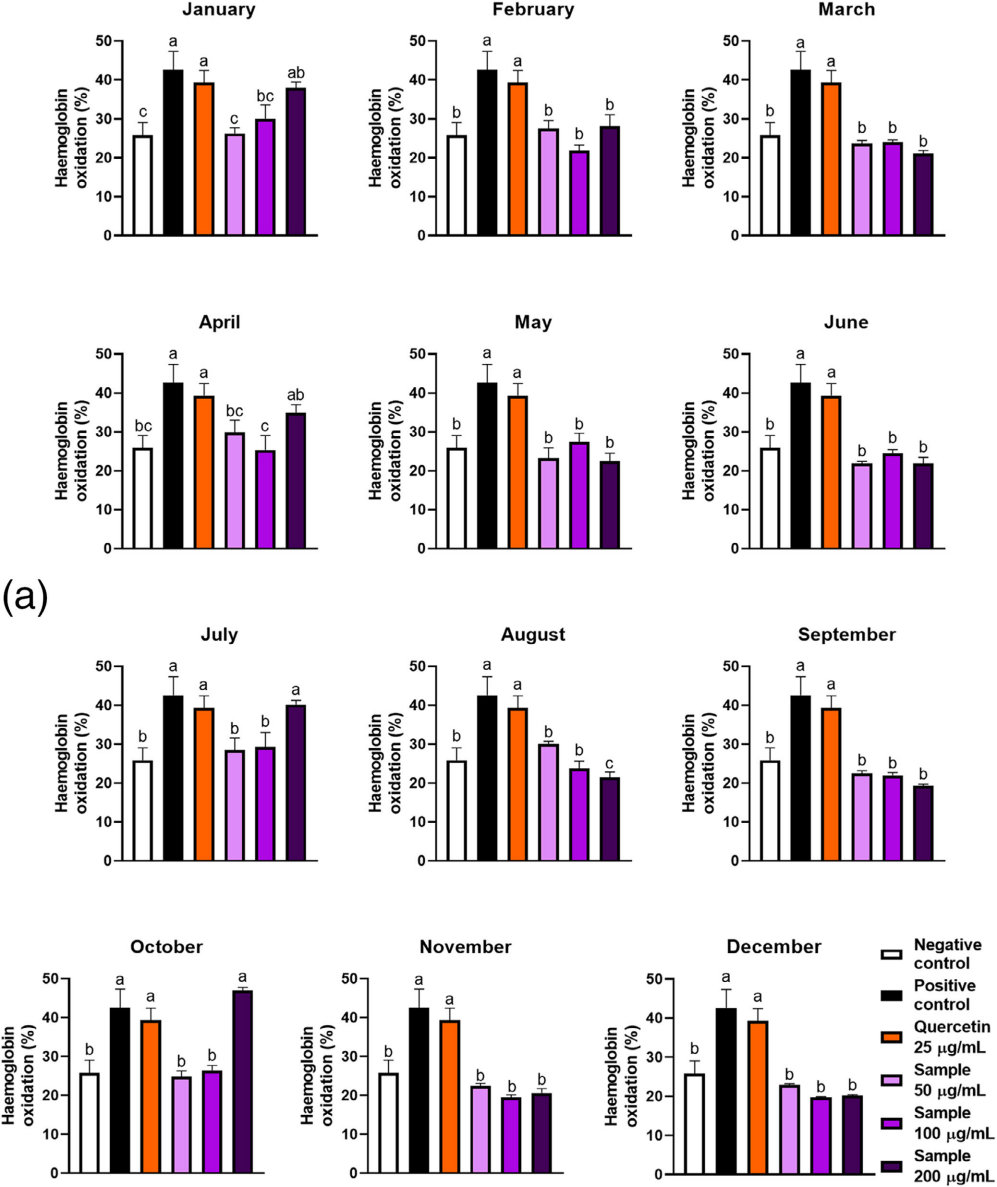

**Figure:**
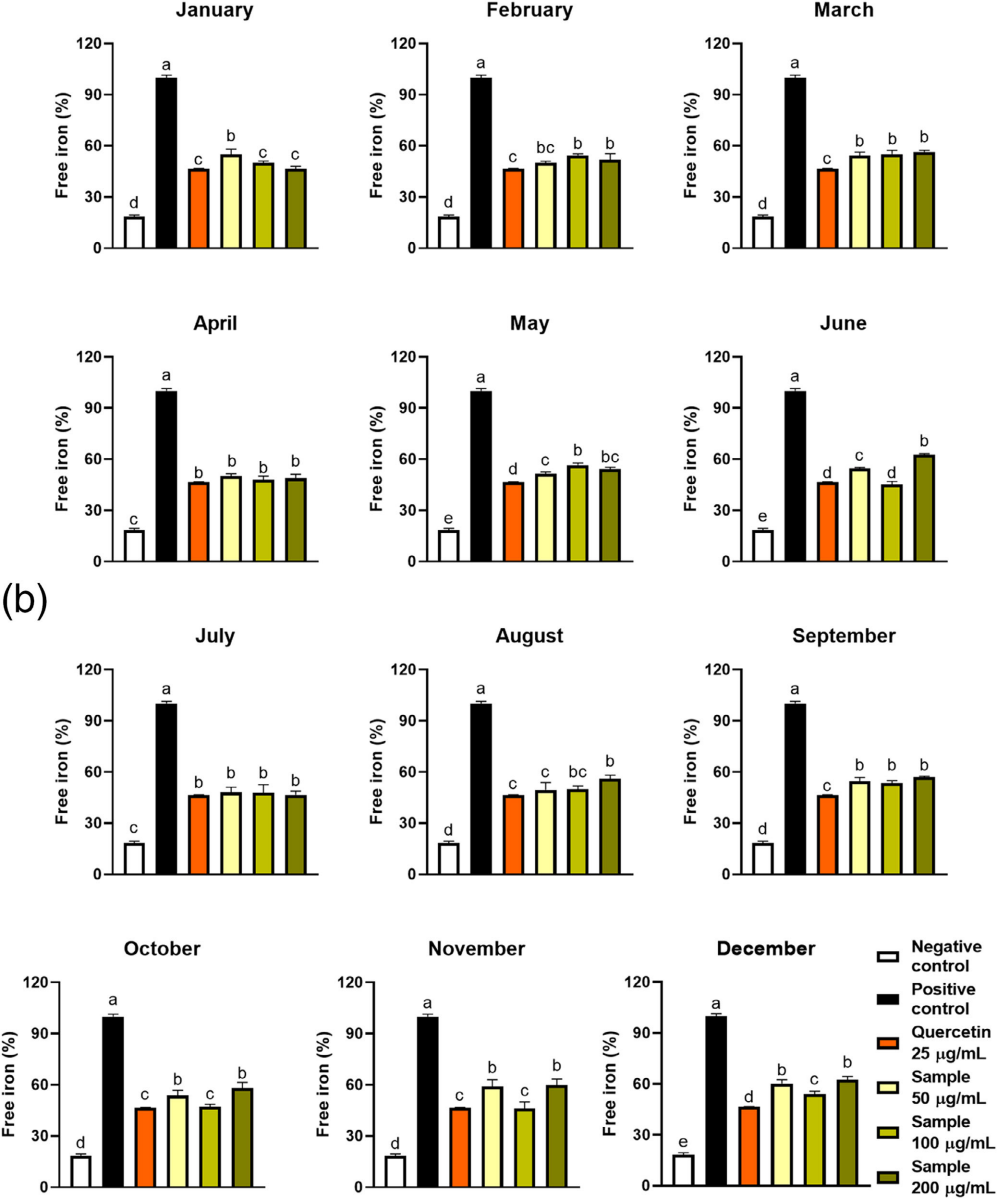

### 2.6 Free Iron

The release of free iron in RBCs occurs as a consequence of the oxidation of some proteins, i.e., haemoglobin, ferritin, and transferrin [34]. In addition to being an oxidative stress marker, free iron may play a prooxidant role in RBC by participating in Fenton reactions and generating more reactive species [35]. To reduce free iron rates, plant extracts may inhibit protein oxidation and/or chelate the iron released from these proteins [10, 34]. The free iron rate in the presence of 100 µg/mL of OPN samples varied between 45% and 56%, all values lower than the positive control (100%) (Table 2). Moreover, only with the February, March, May, September, and December samples, the free iron rate was greater than that verified with quercetin 25 µg/mL. Interestingly, the February, March, April, July, and September samples did not exhibit a dose‐dependent effect (Figure 6B).

## 3 Conclusions

Our results indicated that the polyphenol biosynthesis in OPN leaves may be ruled by environmental conditions, phenological aspects, and seasonal factors. Even rutin, the primary compound of all samples, was detected with different contents depending on the harvest time. This means that OPN responds to external stimuli of biotic or abiotic origin. Protocatechuic acid contents, for example, were correlated with the air relative humidity, solar radiation incidence, and air temperature. More assays are needed to evaluate the real impact of some environmental conditions on the production of these compounds, to maximise their biosynthesis, and to optimise their intake from OPN leaves in the human diet.

Seasonal changes in leaf chemical composition affected RBC protection against oxidative and osmotic stress. These effects were more closely related to individual phenolic compounds than to TPC. Based on Pearson's correlation, ferulic acid, quercetin, protocatechuic acid, and *p*‐coumaric acid seem to be critical compounds to the antioxidant and antihaemolytic effects of OPN leaves, but partitioning studies are required to confirm this. The extracts protected RBC against oxidative and osmotic stresses, despite some samples needing a higher concentration to be effective. Besides, none of the samples exhibited toxic behaviour to the erythrocytes, which suggests that the OPN leaves consumption appears to be safe throughout the year. In the future, a multi‐specimen or multi‐location study is recommended, aiming to exclude the microenvironmental variabilities and guarantee the safety of OPN consumption. These studies may point out which variances are common to a greater number of OPN species and are, in fact, related to the seasonality, and which ones are related to one specimen (i.e. microbiological infection).

In general, consumption safety seems to be consistent throughout the year, but bioactivity varies seasonally. The ideal harvesting time depends on the goal: for the greatest polyphenols intake, September appears to be the best option; to protect erythrocytes against oxidative stress, July, October, and November exhibited the best results.

## 4 Experimental

### 4.1 Chemicals and Plant Material

NaH_2_PO_4_.H_2_O, Na_2_HPO_4_, NaCl, and Folin‐Ciocalteu reagent were bought from Biotec (Curitiba, Brazil). From Vetec (Rio de Janeiro, Brazil), DPPH, methanol, absolute ethanol, ascorbic acid, and disodium ethylenediaminetetraacetic acid monohydrate (EDTA). FeSO_4_.6H_2_O was bought from Neon (São Paulo, Brazil) and bovine serum albumin (BSA), acetonitrile, quercetin, rutin, gallic acid, ferrozine, and the *p*‐coumaric, ferulic, thiobarbituric, and caffeic acids were bought from Sigma‐Aldrich (São Paulo, Brazil), while formic acid was obtained from Reagen (Rio de Janeiro, Brazil). The assays were performed using ultrapure water (Milli‐Q, São Paulo, Brazil). OPN leaves were collected from one specimen located in Francisco Beltrão, Brazil (26°04'51.9''S 53°03'59.5''W) on the first day of each month, between October 2019 and September 2020 (exsiccate number 22500, UEPG Herbarium). The leaves were dried in the dark at room temperature.

### 4.2 Extraction Procedure

Extractions were conducted by infusion at 45°C for 30 min under constant stirring, with a plant material‐solvent ratio of 1:20 (w/v). The solvent was a binary mixture of water and ethanol (60:40 v/v). This solvent composition was chosen because it maximises the extraction of bioactive compounds from OPN leaves, according to the previously optimised conditions by our research group [6]. The extracts were roto‐evaporated and lyophilised (‐50°C, 1200 µmHg; Terroni LD 1500A, São Paulo, Brazil).

## 5 Chemical Composition

### 5.1 Total Phenolic Content

The Folin‐Ciocalteu method was used to quantify the samples’ TPC [36], based on an analytical curve of gallic acid (0–120 mg/L, R^2^ = 0.9987). The results were expressed as mg of gallic acid equivalent per 100 g dry leaves (mg GAE/100 g).

### 5.2 Individual Phenolic Quantification by High‐Performance Liquid Chromatography/Diode Array Detector/Ultraviolet

Individual phenolic compounds were quantified by high‐performance liquid chromatography (HPLC) in a Shimadzu LC‐20T chromatograph, equipped with a diode detector array (DAD), degassing system, and an auto‐sampler, with a chromatographic separation in a reverse phase column (C_18_, 150 mm × 4.6 mm, particle size of 3.5 µm). Chromatographic separation was performed with the column at 40°C, with the injection of 10 µL of the samples (filtered with a 0.22 µm nylon membrane) and 500 µL/min of elution flow rate. The elution gradients were previously proposed by our research group [6], in which the mobile phase corresponds to water acidified with 0.2% (v/v) formic acid and acetonitrile. The compounds were detected at 255, 272, 318, 325, and 360 nm and were quantified through standard curves (R^2^ ≥ 0.995, Table S1) with results expressed in µg/g dry leaves.

### 5.3 Chemical Antioxidant Activity

The DPPH radical scavenging was evaluated by constructing an analytical curve of ascorbic acid of 5–25 mg/L (R^2^ = 0.999), with the results expressed in mg AAE/100 g dry OPN leaves [37]. The samples' FRAP was assessed by using a standard curve built with ascorbic acid (10–60 mg/L, R^2^ = 0.998) and the results were expressed in mg AAE/100 g dry plant material [38]. To investigate Fe^2+^ chelating ability, a standard curve was constructed with EDTA (5–50 mg/L, R^2^ = 0.992) with results expressed in mg EDTAE/100 g dry plant material [39].

### 5.4 Erythrocytes Protection Against Osmotic and Oxidative Stresses

The procedures, performed with an O^+^ type blood sample (female donor) obtained from the Hospital Regional Universitário Wallace Thadeu de Mello e Silva, were duly approved by the Ethics Committee of the State University of Ponta Grossa (CAAE 94830318.1.0000.0105). RBC were submitted to osmotic stress by incubating the cells under sundry different osmotic pressures, by modifying the NaCl concentration [40]. RBC oxidation was induced with AAPH 200 mmol/L at 37°C for 2 h. The negative control (minimal oxidation) was obtained by replacing the sample and AAPH with PBS, while the positive control (maximum oxidation) was obtained by replacing only the sample with PBS. The haemolysis rate, lipoperoxidation [10], and haemoglobin oxidation [29] were evaluated. Yet, free iron was also assessed [41] with slight modifications. 125 µL of hemolysate reacted with 100 µL of ascorbic acid 250 mg/L for 5 min, and then, 75 µL of ferrozine 8 mmol/L was mixed. After 30 min, the absorbances were measured at 562 nm. The results were expressed as free iron releasing rate, calculated using Equation (1), where A_Sample_ is the sample's absorbance and A_Positive control_ is the positive control's absorbance.

(1) $$\mathrm{Free}\mathrm{iron}\mathrm{releasing}\left(\%\right)=\left(A_{\mathrm{Sample}}/A_{\mathrm{Positive}\mathrm{control}}\right)x100$$

### 5.5 Meteorological Data and Phenological Aspects

The Paraná State Technology and Environmental Monitoring System (SIMEPAR) provided meteorological data collected at the Francisco Beltrão SIMEPAR Weather Station (Table S2). The OPN tree phenological aspects were based on those previously reported by Silva et al. [12], and these aspects are shown in Figure S1.

### 5.6 Statistical Analysis

The assays were performed in triplicate, with results expressed as mean and standard deviation. Brown‐Forsythe test for homoscedasticity and one‐dimensional analysis of variance for the difference between means were performed, and Pearson correlation matrices were used to statistically correlate chemical composition, antioxidant activity, and bioactivities. The tests were carried out using the TIBCO Statistica v. 13.3 software (TIBCO Software Inc., Palo Alto, USA), with a significance level of 0.05.

## Author Contributions

**Thiago Mendanha Cruz**: data curation, formal analysis, investigation, visualisation and writing – original draft; **Cristiane de Moura**: formal analysis, investigation and writing – review & editing; **Daniel Granato**: methodology, supervision and writing – review & editing; **Mariza Boscacci Marques**: conceptualisation, methodology, project administration, supervision and writing – original draft.

## Funding

We kindly acknowledge CAPES (Superior Education Personnel Enhancement Coordination) for the master's and doctorate scholarships.

## Conflicts of Interest

The authors declare no conflicts of interest.

## Supporting information




**Supporting File 1**: cbdv70764‐sup‐0001‐SuppMat.docx

## Data Availability

The authors have nothing to report.
