# Supplementary material for: Seasonality Affects the Phenolic Composition and Erythroprotective Activity of Ora‐Pro‐Nobis (Pereskia aculeata) Leaves
Source: Chem Biodivers. 2025 Dec 19;23(2):e02379. doi: 10.1002/cbdv.202502379 (PMC12860515; doi:10.1002/cbdv.202502379)

Table S1 – Retention time (t_R_), wavelength used to detect, analytical curve, and limits of detection (LOD) and quantification (LOQ) for each phenolic compound investigated in ora-pro-nobis leaves by HPLC-DAD-UV.

| **Peak** | **t_R_ (min)** | **Compound** | **Detection λ (nm)** | **Analytical curve** | **R²** | **LOD (mg/L)** | **LOQ (mg/L)** |
| --- | --- | --- | --- | --- | --- | --- | --- |
| 1 | 7.72 | Gallic acid | 272 | y = 56600x + 24052 | 0.998 | 0.018 | 0.055 |
| 2 | 15.00 | Protocatechuic acid | 255 | y = 62841x – 24604 | 0.999 | 0.017 | 0.052 |
| 3 | 17.08 | Rutin | 360 | y = 35743x – 1028 | 0.999 | 0.036 | 0.111 |
| 4 | 19.52 | Quercetin | 360 | y = 76546x – 7062 | 0.999 | 0.010 | 0.031 |
| 5 | 26.66 | Caffeic acid | 325 | y = 105042x – 53002 | 0.997 | 0.006 | 0.018 |
| 6 | 29.68 | *p*-Coumaric acid | 318 | y = 124348x + 142935 | 0.995 | 0.219 | 0.503 |
| 7 | 30.15 | Ferulic acid | 325 | y = 105233x – 17124 | 0.999 | 0.041 | 0.125 |

Table S2 – Report of the Paraná Weather System (SIMEPAR) of data from the Francisco Beltrão Weather Station (PR).

| **Sample** | **Daily solar radiation (W/m^2^)** | | | | **Daily air temperature (ºC)** | | | | **Precipitation (mm)** | | | **Relative Air Humidity (%)** | | | |
| --- | --- | --- | --- | --- | --- | --- | --- | --- | --- | --- | --- | --- | --- | --- | --- |
|  | **Monthly** | | | **Harvest day** | **Monthly** | | | **Harvest day** | **Monthly** | | **Harvest day** | **Monthly** | | | **Harvest day** |
|  | **Min.** | **Max.** | **Average** |  | **Min.** | **Max.** | **Average** |  | **Max.** | **Average** |  | **Min.** | **Max.** | **Average** |  |
| Oct/2019 | 122.398 | 553.204 | 395.523 | 511.606 | 16.503 | 27.393 | 22.422 | 25.696 | 47.6 | 121.4 | 0.0 | 45.380 | 94.027 | 73.071 | 45.380 |
| Nov/2019 | 88.143 | 575.589 | 412.451 | 543.387 | 19.878 | 25.759 | 22.878 | 23.726 | 39.6 | 161.4 | 0.0 | 60.065 | 99.768 | 77.196 | 74.088 |
| Dec/2019 | 105.679 | 603.889 | 415.432 | 459.306 | 18.603 | 26.243 | 22.493 | 24.236 | 39.6 | 134.6 | 0.0 | 60.193 | 99.449 | 77.677 | 68.796 |
| Jan/2020 | 155.394 | 559.685 | 393.908 | 343.270 | 20.576 | 25.533 | 23.799 | 24.229 | 47.4 | 189.2 | 0.0 | 65.314 | 97.573 | 79.664 | 86.264 |
| Feb/2020 | 100.727 | 582.930 | 447.121 | 565.500 | 18.419 | 25.910 | 23.274 | 24.323 | 76.0 | 152.6 | 0.0 | 61.847 | 99.656 | 74.081 | 71.955 |
| Mar/2020 | 36.472 | 540.996 | 478.203 | 522.069 | 19.826 | 25.649 | 22.731 | 22.666 | 55.4 | 60.2 | 0.0 | 55.594 | 97.943 | 69.728 | 59.468 |
| Apr/2020 | 42.752 | 506.630 | 421.013 | 443.036 | 13.318 | 24.097 | 18.351 | 24.097 | 28.2 | 41.4 | 0.0 | 55.559 | 95.370 | 71.943 | 73.146 |
| May/2020 | 37.732 | 435.455 | 310.974 | 119.118 | 9.945 | 19.659 | 14.486 | 17.751 | 93.0 | 241.0 | 0.8 | 70.144 | 100.000 | 81.736 | 95.997 |
| Jun/2020 | 41.816 | 368.429 | 241.806 | 62.635 | 10.890 | 22.492 | 16.335 | 14.124 | 47.4 | 217.2 | 4.0 | 72.077 | 100.000 | 89.758 | 99.929 |
| Jul/2020 | 91.484 | 407.907 | 297.626 | 201.150 | 7.089 | 19.656 | 14.624 | 9.617 | 18.8 | 51.0 | 0.0 | 71.246 | 99.311 | 84.401 | 83.877 |
| Aug/2020 | 20.039 | 476.724 | 345.947 | 412.012 | 5.989 | 21.969 | 16.460 | 15.660 | 69.4 | 149.8 | 0.0 | 56.564 | 100.000 | 78.309 | 83.773 |
| Sep/2020 | 154.250 | 498.433 | 375.760 | 425.033 | 16.881 | 27.270 | 21.120 | 20.775 | 10.6 | 24.4 | 0.0 | 54.722 | 96.414 | 72.619 | 78.426 |

Table S3 – Pearson’s correlation matrices between the phenolic composition and the chemical antioxidant and hemoprotector activities.

|  | **DPPH** | **FRAP** | **Fe^2+^**  **chelating** | **Hypotonic**  **hemolysis** | **H_50_** | **AAPH-induced**  **hemolysis** | **Hemoglobin**  **oxidation** | **RBC TBARS** | **Free**  **iron** |
| --- | --- | --- | --- | --- | --- | --- | --- | --- | --- |
| **Gallic acid** | *r* = -0.309  *p* = 0.328 | *r* = -0.419  *p* = 0.176 | *r* = -0.350  *p* = 0.264 | *r* = -0.220  *p* = 0.491 | *r* = 0.008  *p* = 0.981 | *r* = -0.093  *p* = 0.773 | *r* = -0.137  *p* = 0.671 | *r* = 0.383  *p* = 0.220 | *r* = 0.464  *p* = 0.129 |
| **Protocatechuic**  **acid** | *r* = 0.564  *p* = 0.056 | *r* = -0.021  *p* = 0.949 | *r* = 0.803  *p* = 0.002 | *r* = 0.060  *p* = 0.853 | *r* = -0.368  *p* = 0.239 | *r* = 0.388  *p* = 0.212 | *r* = -0.649  *p* = 0.022 | *r* = -0.226  *p* = 0.481 | *r* = -0.223  *p* = 0.486 |
| **Caffeic acid** | *r* = 0.273  *p* = 0.390 | *r* = -0.347  *p* = 0.270 | *r* = 0.499  *p* = 0.098 | *r* = -0.106  *p* = 0.743 | *r* = -0.346  *p* = 0.270 | *r* = 0.378  *p* = 0.226 | *r* = -0.381  *p* = 0.221 | *r* = -0.285  *p* = 0.482 | *r* = -0.421  *p* = 0.172 |
| ***p*-Coumaric acid** | *r* = 0.139  *p* = 0.666 | *r* = -0.162  *p* = 0.615 | *r* = 0.647  *p* = 0.023 | *r* = 0.152  *p* = 0.638 | *r* = -0.007  *p* = 0.984 | *r* = 0.343  *p* = 0.276 | *r* = -0.653  *p* = 0.021 | *r* = 0.013  *p* = 0.967 | *r* = -0.021  *p* = 0.949 |
| **Ferulic acid** | *r* = 0.472  *p* = 0.121 | *r* = 0.201  *p* = 0.531 | *r* = 0.195  *p* = 0.544 | *r* = -0.155  *p* = 0.631 | *r* = -0.188  *p* = 0.559 | *r* = 0.258  *p* = 0.417 | *r* = -0.211  *p* = 0.510 | *r* = -0.737  *p* = 0.006 | *r* = -0.466  *p* = 0.127 |
| **Rutin** | *r* = 0.251  *p* = 0.430 | *r* = 0.037  *p* = 0.909 | *r* = 0.408  *p* = 0.188 | *r* = 0.316  *p* = 0.317 | *r* = 0.168  *p* = 0.602 | *r* = 0.309  *p* = 0.329 | *r* = -0.300  *p* = 0.343 | *r* = 0.093  *p* = 0.775 | *r* = -0.081  *p* = 0.803 |
| **Ellagic acid** | *r* = 0.055  *p* = 0.866 | *r* = 0.057  *p* = 0.875 | *r* = 0.093  *p* = 0.773 | *r* = 0.334  *p* = 0.289 | *r* = 0.277  *p* = 0.384 | *r* = -0.397  *p* = 0.202 | *r* = 0.303  *p* = 0.339 | *r* = -0.323  *p* = 0.306 | *r* = -0.363  *p* = 0.247 |
| **Quercetin** | *r* = -0.032  *p* = 0.921 | *r* = 0.240  *p* = 0.452 | *r* = 0.548  *p* = 0.065 | *r* = -0.596  *p* = 0.041 | *r* = -0.531  *p* = 0.076 | *r* = 0.079  *p* = 0.808 | *r* = -0.375  *p* = 0.230 | *r* = -0.218  *p* = 0.497 | *r* = -0.087  *p* = 0.789 |
| **TPC** | *r* = 0.741  *p* = 0.006 | *r* = 0.147  *p* = 0.649 | *r* = 0.861  *p* = <0.001 | *r* = 0.044  *p* = 0.891 | *r* = -0.247  *p* = 0.440 | *r* = 0.522  *p* = 0.082 | *r* = -0.676  *p* = 0.016 | *r* = -0.256  *p* = 0.422 | *r* = -0.119  *p* = 0.712 |

Figure S1 – Phenological aspects of OPN grown in Brazil.


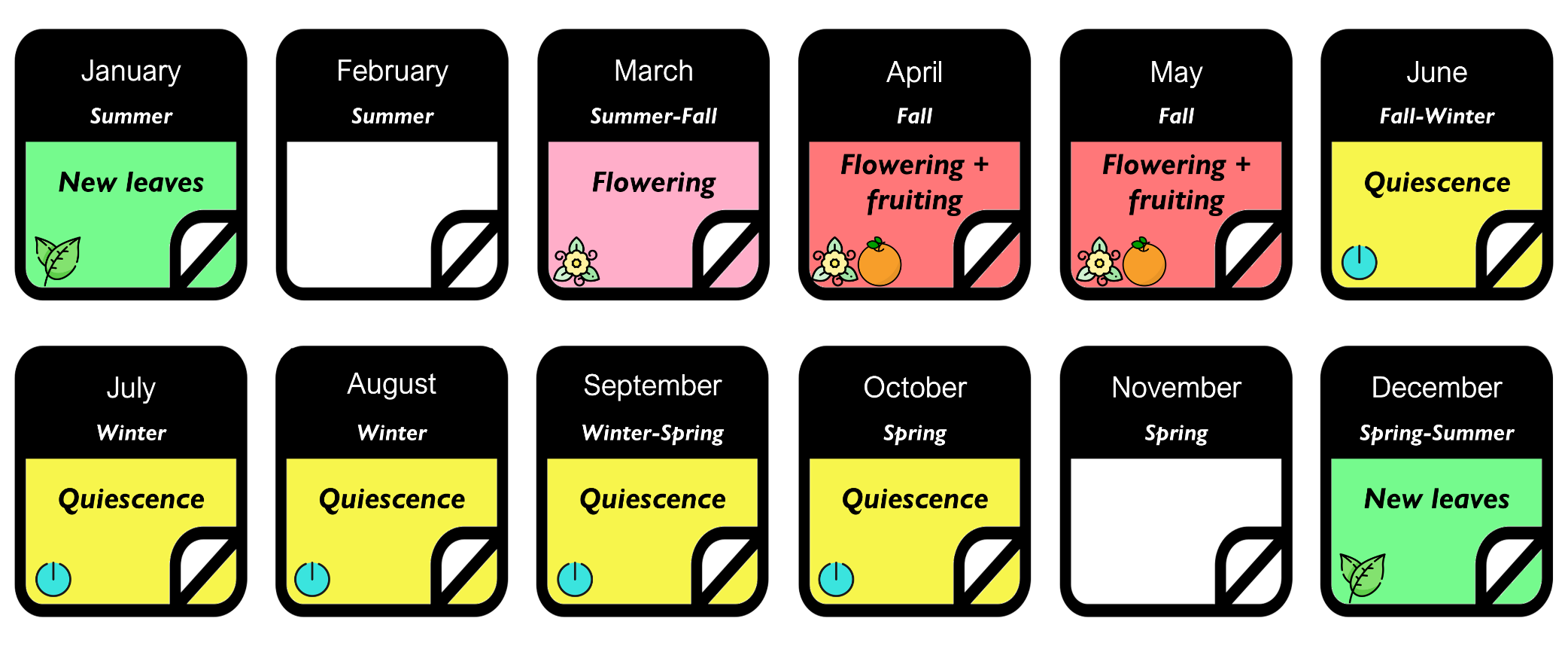


Figure S2 – Chromatograms of phenolic compounds detected in OPN leaves extracts. The numbering of peaks is described in Table S1.


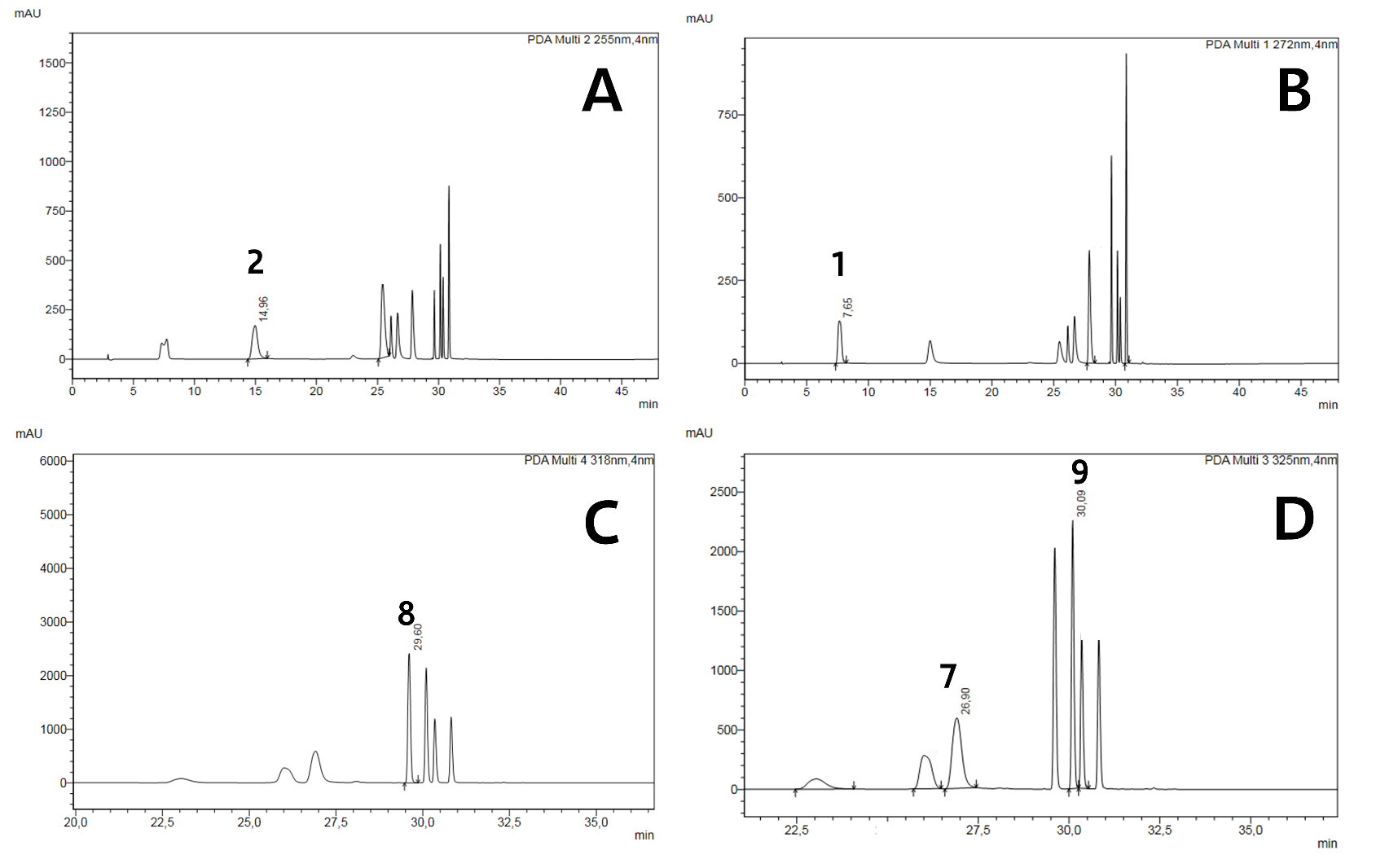


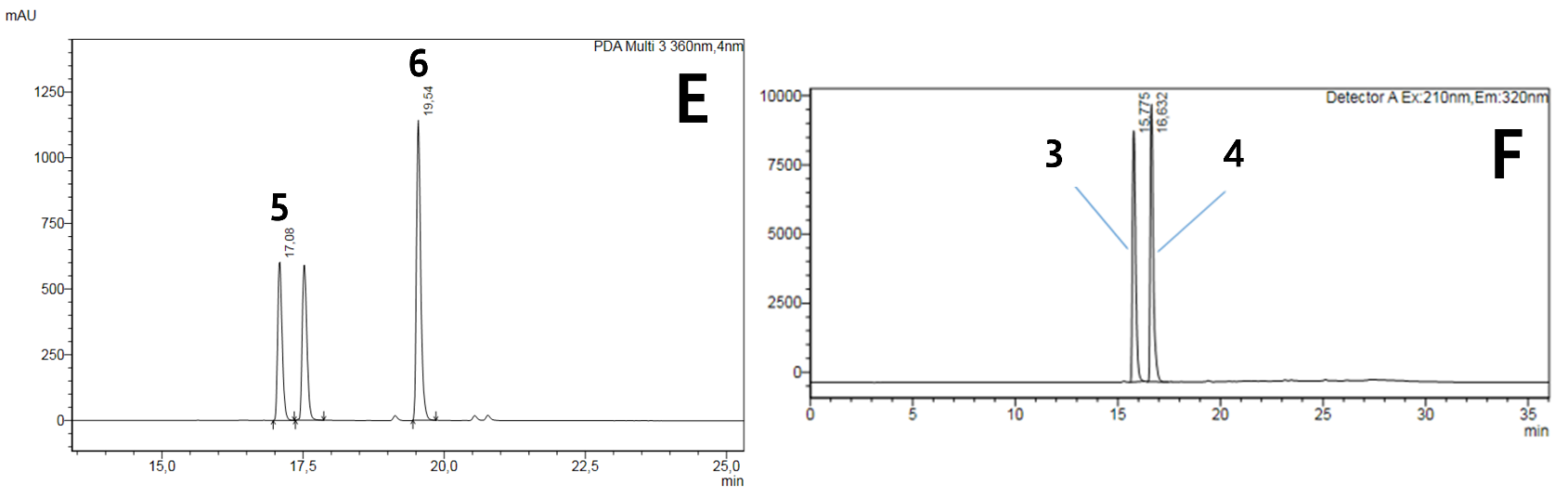


Figure S3 – Fluctuation of the individual phenolic content of OPN leaf extracts throughout the year.


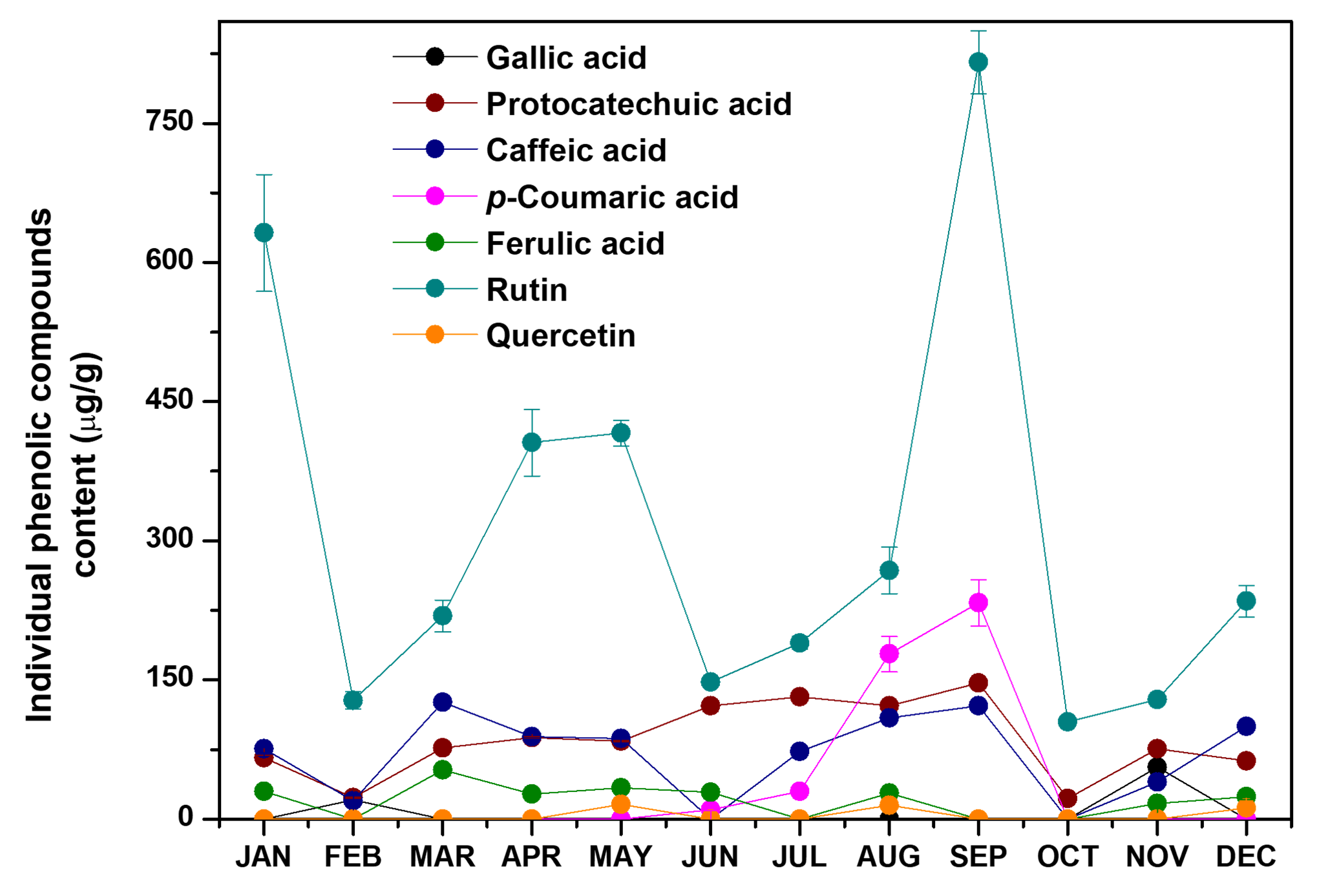


Figure S4 – Fluctuation of the chemical antioxidant activity of OPN leaf extracts throughout the year.


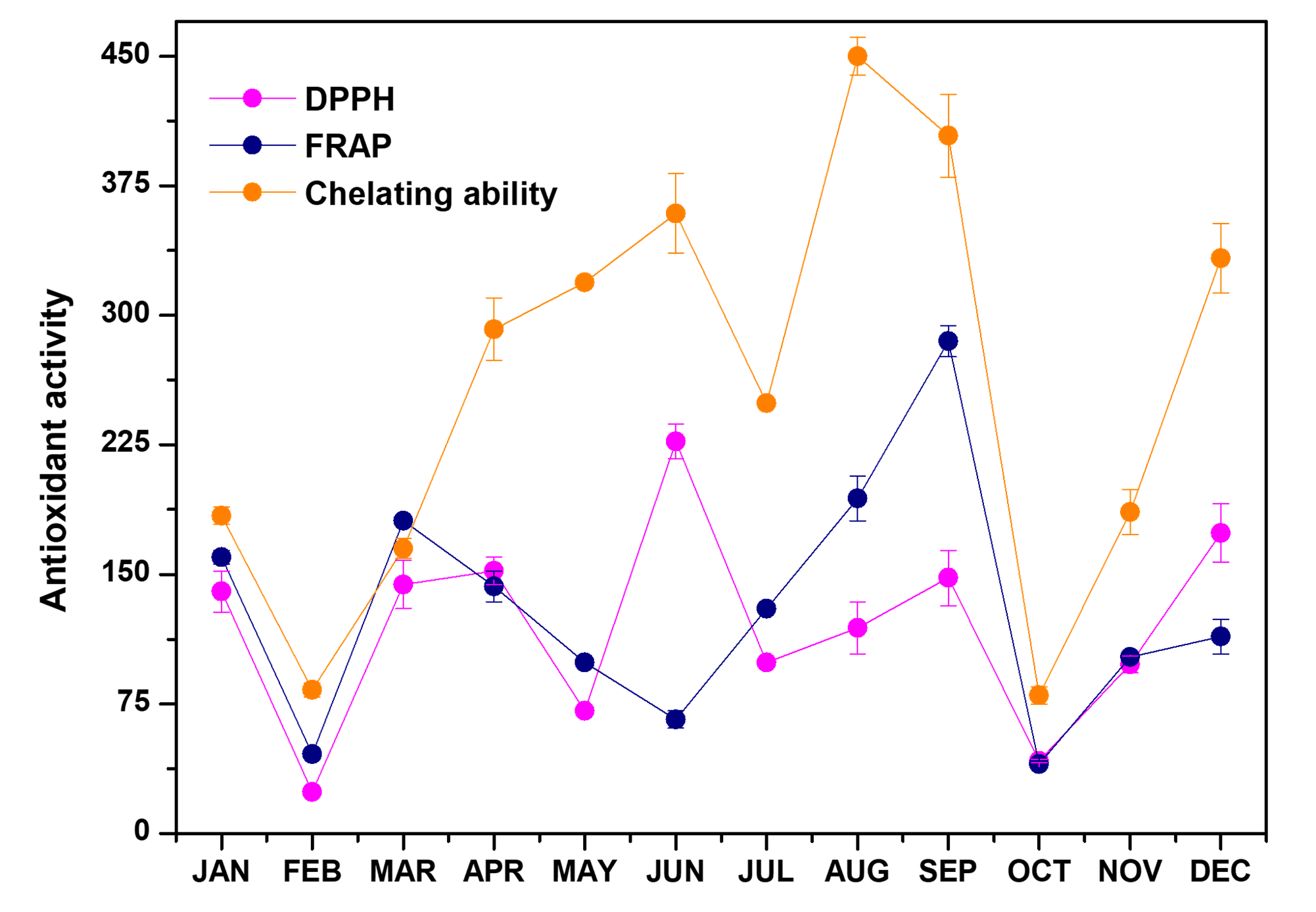

Supplement: Supplementary file 1 — Supporting File 1: cbdv70764‐sup‐0001‐SuppMat.docx [file CBDV-23-e02379-s001.docx]
